# RNA interference for apoptosis signal-regulating kinase-1 (ASK-1) rescues photoreceptor death in the rd1 mouse

**Published:** 2009-09-02

**Authors:** Daisuke Sekimukai, Shigeru Honda, Akira Negi

**Affiliations:** Department of Surgery, Division of Ophthalmology, Kobe University Graduate School of Medicine, Kobe, Japan

## Abstract

**Purpose:**

To evaluate whether RNA interference against apoptosis signal-regulating kinase-1 (*ASK-1*), a gene involved in stress-induced apoptosis, inhibits photoreceptor death in retinal degeneration 1 (rd1) mice.

**Methods:**

Retinal explants from rd1 mice were subjected to organ cultures on postnatal day 9 (P9). Short interfering RNA (siRNA) for *ASK-1* was transfected into cultured retinas at the onset of experiments. Real-time PCR was performed to evaluate the natural expression of *ASK-1* mRNA and its inhibition with siRNA. Retinal explants were fixed at P13 and P16, and consecutive cryosections were prepared. Histological and immunohistochemical examinations including TUNEL assays were performed.

**Results:**

In preliminary experiments, the incorporation of fluorescent siRNA was found in cells in the outer nuclear and inner nuclear layers on the day following transfection. The expression of *ASK-1* mRNA increased with time, which was suppressed more than 70% by siRNA. ASK-1 immunopositive cells were found mostly in the outer nuclear layers, and the number of immunopositive cells was remarkably reduced in retinas treated with siRNA for *ASK-1* compared to untreated controls. The thickness of outer nuclear layers of control retinas decreased with time, while the thickness of siRNA transfected retinas was significantly preserved compared to control at P16 (p=0.0021). In TUNEL assays, siRNA for *ASK-1* significantly decreased TUNEL-positive cells (49% and 42% of controls at P13 and 16, p=0.039 and 0.0028, respectively).

**Conclusions:**

RNA interference against *ASK-1* may provide a benefit by inhibiting photoreceptor apoptosis in rd1 mice.

## Introduction

Retinitis pigmentosa (RP) is a major retinal hereditary disease characterized by progressive degeneration of photoreceptors and retinal pigment epithelial cells (RPEs). RP causes night blindness, visual field contraction, and eventually, severe visual disturbance [1]. Studies have disclosed several mutations in photoreceptor and RPE genes that associate with its pathogenesis. At least 30 genes have been identified, many of which encode photoreceptor-specific proteins such as peripherin [2], rod outer segment membrane protein 1 [3], rod cyclic GMP (cGMP) phosphodiesterase [4], and rhodopsin [5]. Animal models of retinal degeneration are widely used to investigate genetic mechanisms for photoreceptor apoptosis, a feature common to all cases of human RP. In the retinal degeneration 1 (rd1) mouse, a mutation located in the gene encoding the beta-subunit of rod cGMP phosphodiesterase [6] causes accumulation of cGMP followed by an increase in calcium ion (Ca^2+^) influx to the cytoplasm, which activates Ca^2+^ dependent proteases, such as calpain and cathepsin D, or causes mitochondrial stress, which induces apoptosis of photoreceptors early in postnatal development [7,8]. Since mutations in this gene are the most common cause of autosomal recessive RP in humans [4], it is a particularly relevant model. Research has demonstrated that multiple pathways exist in the process of photoreceptor apoptosis in the rd1 mouse [9-11].

Apoptosis signal-regulating kinase 1 (*ASK-1*) is a mitogen-activated protein (MAP) kinase family member, which is one of the activators of the p38-JNK apoptosis signaling cascade [12,13]. ASK-1 is activated in response to various cytotoxic stresses, including TNF, Fas, and reactive oxygen species (ROS) such as hydrogen peroxide (H_2_O_2_) [13-16]. Overexpression of wild-type or constitutively active ASK1 induces apoptosis in various cells through mitochondria-dependent caspase activation [13,15,16], and ASK1 is required for apoptosis induced by oxidative stress, TNF and endoplasmic reticulum (ER) stress [17,18]. Moreover, reports showed that Ca^2+^ signaling regulates the ASK1–p38-MAP kinase cascade [19]. Ca^2+^ influx evoked by membrane depolarization in primary neurons and synaptosomes induced activation of p38, which was impaired in samples derived from ASK1-deficient mice. However, to date, no study has addressed the role of ASK-1 in photoreceptor apoptosis in the rd1 mouse. We investigated the expression and distribution of ASK-1 in retinas from rd1 mice.

RNA interference is a technology that can knock down the expression of specific genes using a few copies of short RNA [20]. This is known to be quite effective in suppressing genes in vitro, but few reports have been published about the use of RNA interference in vivo or in organ cultures [21-23]. In this study, we evaluated whether RNA interference for *ASK-1* works in retinal organ cultures and inhibits photoreceptor death in rd1 mice.

## Methods

### Retinal organ cultures

The study adhered to the Association for Research in Vision and Ophthalmology (ARVO) Statement for the Use of Animals in Ophthalmic and Vision Research. Retinal explant cultures of C3H/HeN (rd1) mice and C57Bl/6 (Bl6) mice were prepared as described previously [24]. The mice were purchased from (CLEA Japan Inc., Tokyo, Japan) and kept in mouse cages with free access of solid food and water. Light-dark cycle was 12:12 h. Briefly, mice were euthanized (intraperitoneal injection of 15 mg of pentobarbital per mouse for euthanasia) and neural retinas were extracted on postnatal day (P) 8 or 9, depending on the experiment. Each retina was submerged in HBSS and extended to make whole flat-mounts on 30 mm diameter microporous membranes (Millicell-CM; Millipore, Bedford, MA) with ganglion cell layers facing up. Millicell-CM containing retinal explants were soaked in 1 ml of culture medium (Opti-MEM, Invitrogen, Carlsbad, CA) with or without 25% heat inactivated horse serum in six-well plates and incubated, allowing an air interface with the ganglion cell side of the retina in a humidified atmosphere of 5% CO_2_ and 95% air at 34 °C. Culture media was changed every other day.

### Transfection of siRNA

For preliminary experiments, to confirm that siRNA was properly incorporated into retinal explants, 100 pmol of fluorescently labeled dsRNA (Block-it^TM^ Alexa Fluor Red Fluor. Oligo, Invitrogen) was bound with 5 μl lipofectamine (RNAiMAX, Invitrogen) in a total 250 μl of serum free culture media for 20 min and applied to retinal organ cultures. After incubation for 4 h at 34 °C, the transfection media was replaced with Opti-MEM culture media containing 25% serum. After 24 h, organ culture cryosections were prepared with DAPI staining and observed with a Keyence Biozero fluorescence microscope (BZ-8000; Keyence, Osaka, Japan). For silencing *ASK-1*, Stealth RNAi, which included two different sequences of siRNA for *ASK-1* (Table 1), were obtained from Invitrogen. Each siRNA was transfected into the retina with lipofectamine RNAiMAX using the same method as described above. Medium GC content scrambled control siRNA (Invitrogen; proprietary sequence) was also applied to examine off-target effects. Retinal explants were either transfected or untransfected with scrambled siRNA and cultured for 72 h before RNA extractions.

**Table 1:** Sequences of Stealth siRNAs for *ASK-1* used in the study.

**Title**	**Lot No.**	**Sequence**
siRNA-1	MSS218535	5′-AAUUGCAGUCUGCACAGCCUUUCGG-3′
siRNA-2	MSS218536	5′-AAAUGCGUAAUGAAACUUCACGUGG-3′

### Real-time PCR

Total RNA was extracted using RNeasy Plus (Qiagen, Valencia, CA) from organ cultures for 0, 1, 3, 5, and 7 days according to the manufacturer’s recommendations. Total RNA was eluted from columns in 50 μl RNase-free water. The purity and concentration of RNA was determined by measuring the absorbance at 260 nm and 280 nm. RNA was reverse-transcribed into cDNA (High Capacity cDNA Reverse Transcription Kit, Applied Biosystems, Foster City, CA) in a total volume of 100 μl according to the manufacturer’s instructions. Real-time PCRs were performed in 96 well plates on an ABI Prism 7500 Sequence Detection System (Applied Biosystems StepOne^TM^ and StepOnePlus^TM^ Real Time PCR System and Taqman Fast Universal PCR Master Mix, Applied Biosystems) according to the manufacturer’s instructions. Final reaction volumes were 25 µl. Each sample was analyzed in triplicate. Thermal cycler conditions were as follows: 2 min at 50 °C, 10 min at 95 °C, followed by 40 cycles of 15 s at 95 °C and 1 min at 60 °C. Sequence Detector Software (Applied Biosystems) was used to extract PCR data, which were exported into Excel 2003 (Microsoft Corporation, Redmond, WA) for further analyses. The amount of targeted gene expressed was normalized to an endogenous reference and relative to a calibrator. *β-Actin* was used as an endogenous reference in these experiments. The formula ΔΔ^CT^ was used to calculate the amount of target gene expression normalized to the endogenous control and relative to the calibrator.

### Histological procedures

After transfections and organ cultures for 0, 4, and 7 days (P9, 13 and 16, respectively), retinal explants were fixed with 4% paraformaldehyde. Cryosections were prepared and stained with DAPI so the thickness of the outer nuclear layer (ONL) could be measured. For immunohistochemistry, sections were blocked with 5% normal goat serum in 0.1 M phosphate buffered saline (PBS; 137 mM NaCl, 2.7 mM KCl, 10 mM Sodium Phosphate dibasic, 2 mM Potassium Phosphate monobasic and a pH of 7.4), and incubated in 1:200 diluted rabbit anti-human ASK-1 antibody (catalog# sc-7931; Santa Cruz Biotech, Santa Cruz, CA), with normal goat serum overnight at 4 °C. This rabbit ASK-1 antibody is compatible with mouse ASK-1. For secondary antibody, incubation with 1:1,000 diluted sheep anti-rabbit IgG (whole molecule), F(ab′)_2_ fragment–Cy3 antibody (Sigma-Aldrich, Tokyo, Japan) was applied for 30 min at room temperature. Nuclei were stained with 1:1,500 diluted TO-PRO-3 iodide (642/661; Invitrogen), and sections were examined using a LSM 5 Pascal confocal imaging system (Carl Zeiss Inc., Tokyo, Japan).

### Terminal dUTP Nick End Labeling

Cryosections were incubated in 50 μl of reaction buffer containing terminal deoxynucleotidyl transferase (TdT; Promega, Southampton, UK) and fluorescein-12-dUTP (Roche, Basel, Switzerland) according to the manufacturer’s instructions. Sections were incubated at 37 °C for 1 h in a humidified chamber. After several washes in PBS and staining with DAPI, sections were observed with a Keyence Biozero fluorescence microscope. Under masked conditions, at least 500 cells in the ONL were counted using digital images at 200× magnification for each condition and the proportion of TUNEL-positive cells was determined.

### Statistical analyses

Unpaired two-tailed Student’s *t*-tests were used for all statistical analyses in the present study. A p value less than 0.05 was considered statistically significant.

## Results

### Incorporation of siRNA

**Figure 1:**
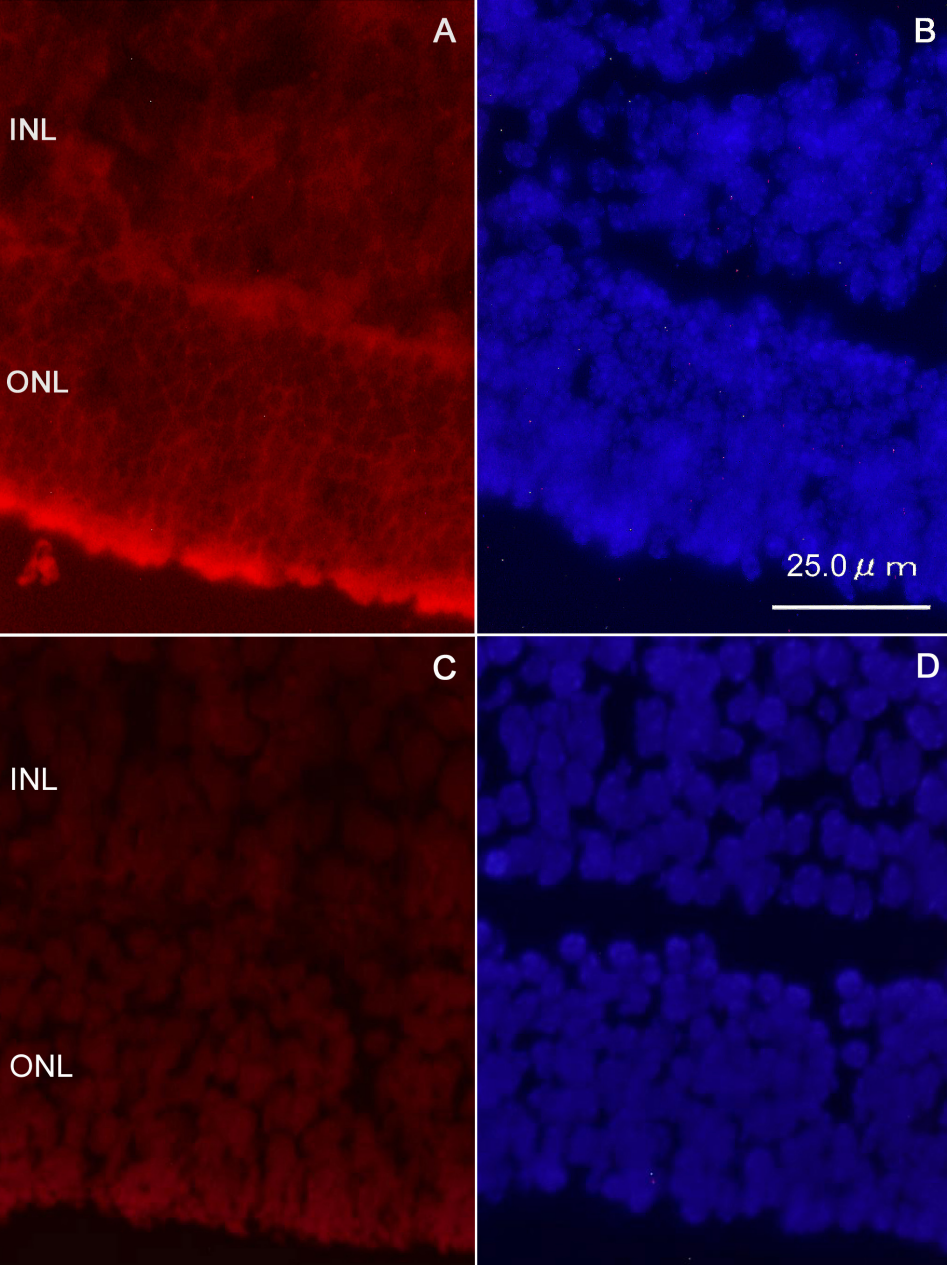
Incorporation of siRNA into retinal explants. **A**: Alexa Fluor red-labeled dsRNA oligonucleotides were found in most cells in outer nuclear layers (ONLs) and inner nuclear layers (INLs) 24 h after transfection. **B**: DAPI staining shows the nuclei in the same section as in **A**. **C**: Image of negative control dsRNA without fluorescent labeling shows no staining. **D**: DAPI staining shows the nuclei in the same section as in **C**.

Fluorescently labeled dsRNA oligonucleotides were found in most cells in ONLs and INLs 24 h after transfections (Figure 1). Labeled oligonucleotides were found only in cytoplasm and not in nuclei.

### Expression of *ASK-1* mRNA

**Figure 2:**
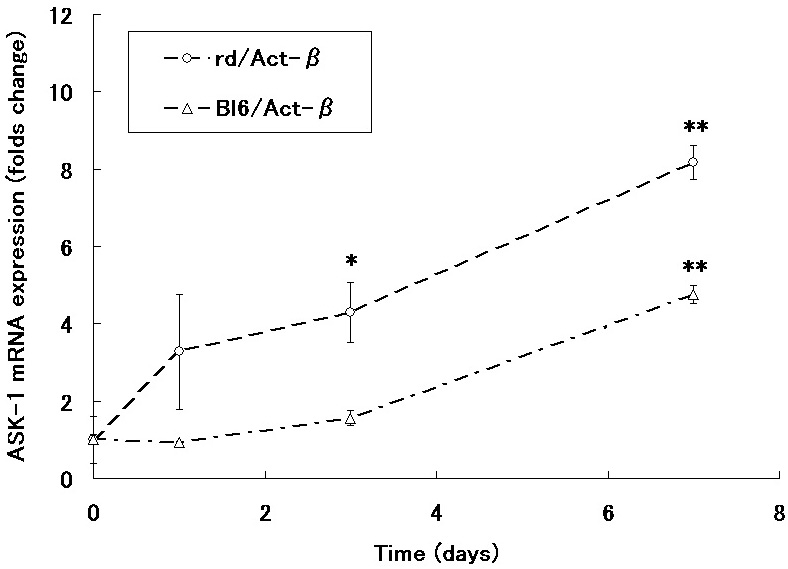
*ASK-1* expression in the retina of rd1 mice. The expression of *ASK-1* mRNA in rd1 mice increased gradually during days 0–7 (corresponds to P8–15). The expression of *ASK-1* mRNA in the retina of Bl6 mice was low until P11 and mildly increased by P15. Values are presented as averages of 3 independent experiments. Asterisk (*) indicates p<0.005, and double asterisk (**) represents a p<0.0005 compared with baseline.

The expression of *ASK-1* mRNA in extracted retinas from rd1 mice increased in a time-dependent manner during experimental days 0 to 7 (corresponds to P8 to P15). Upregulation was significant on days 3 and 7 (Figure 2). The expression of *ASK-1* mRNA in retinas from Bl6 mice was low until day 3 but significantly increased by day 7.

### RNA interference for *ASK-1*

**Figure 3:**
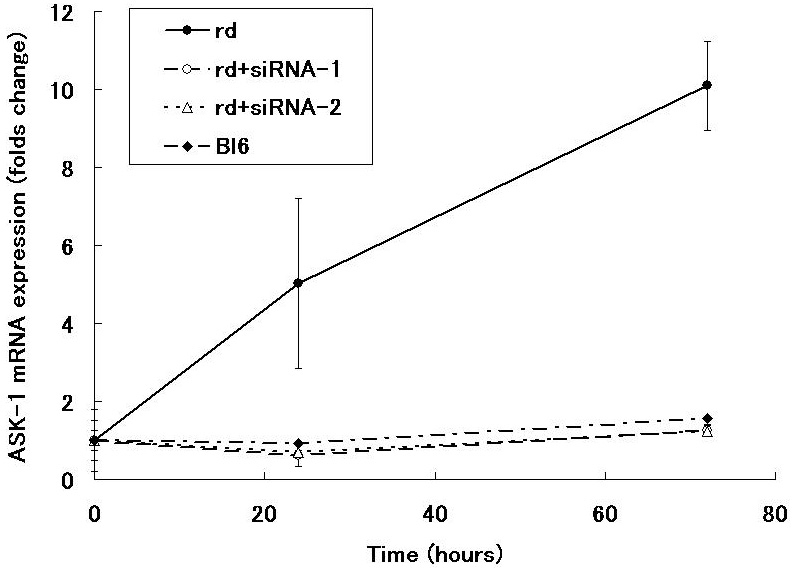
Suppression of *ASK-1* mRNA expression by siRNA. The expression of *ASK-1* mRNA in the retina of rd1 mice was inhibited at 24 and 72 h after the transfection of siRNA. The expression of *β-actin* was used as an internal control. Values are presented as the average±SEM of three independent experiments.

Since the effect of RNA interference is usually evaluated within 72 h [25-27], we determined the relative expression of *ASK-1* mRNA in rd1 mice retinas at 24 h (P9) and 72 h (P11) after siRNA transfection. The expression of *ASK-1* mRNA was suppressed more than 70% with each of two siRNA at both time points (Figure 3). Because of the equal inhibitory effect of both siRNAs, we used siRNA-2 for the subsequent experiments. In the control experiment, retinal explants treated with scrambled siRNA showed higher expression of *ASK-1* (3.5 fold compared to starting point, n=3) than untreated subjects (threefold compared to starting point, n=3) at 72 h of incubation although the difference was not significant.

### Immunoreactivity of ASK-1

**Figure 4:**
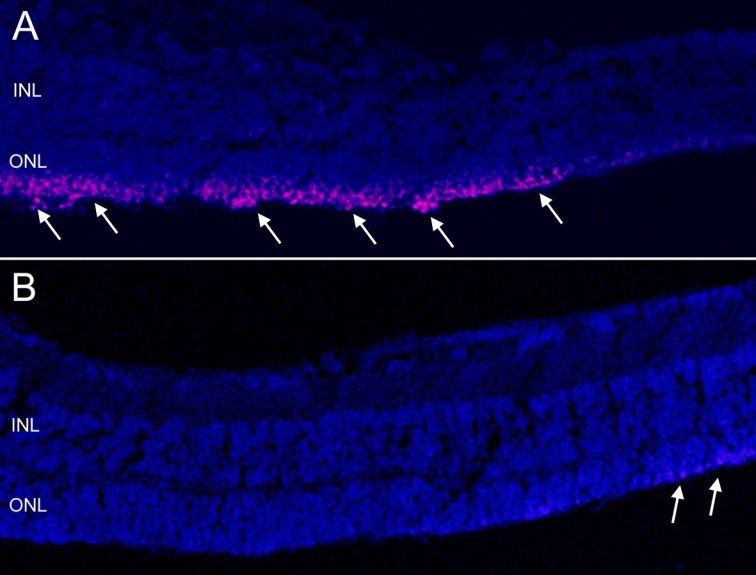
Immunohistochemistry of ASK-1 on experimental day 4. **A**: Immunoreactivity of ASK-1 was found in the outer part of ONL from control retina in rd1 mice (arrows). **B**: Immunoreactivity was weakly found (arrows) in retina transfected with *ASK-1* siRNA. Images are shown at 200× magnification.

Immunoreactivity of ASK-1 was found in the outer part of ONLs of control retinas (day 4 corresponding to P12; Figure 4). However, the immunoreactivity was very weak for the same time period with transfected siRNA-2.

### Histological findings

**Figure 5:**
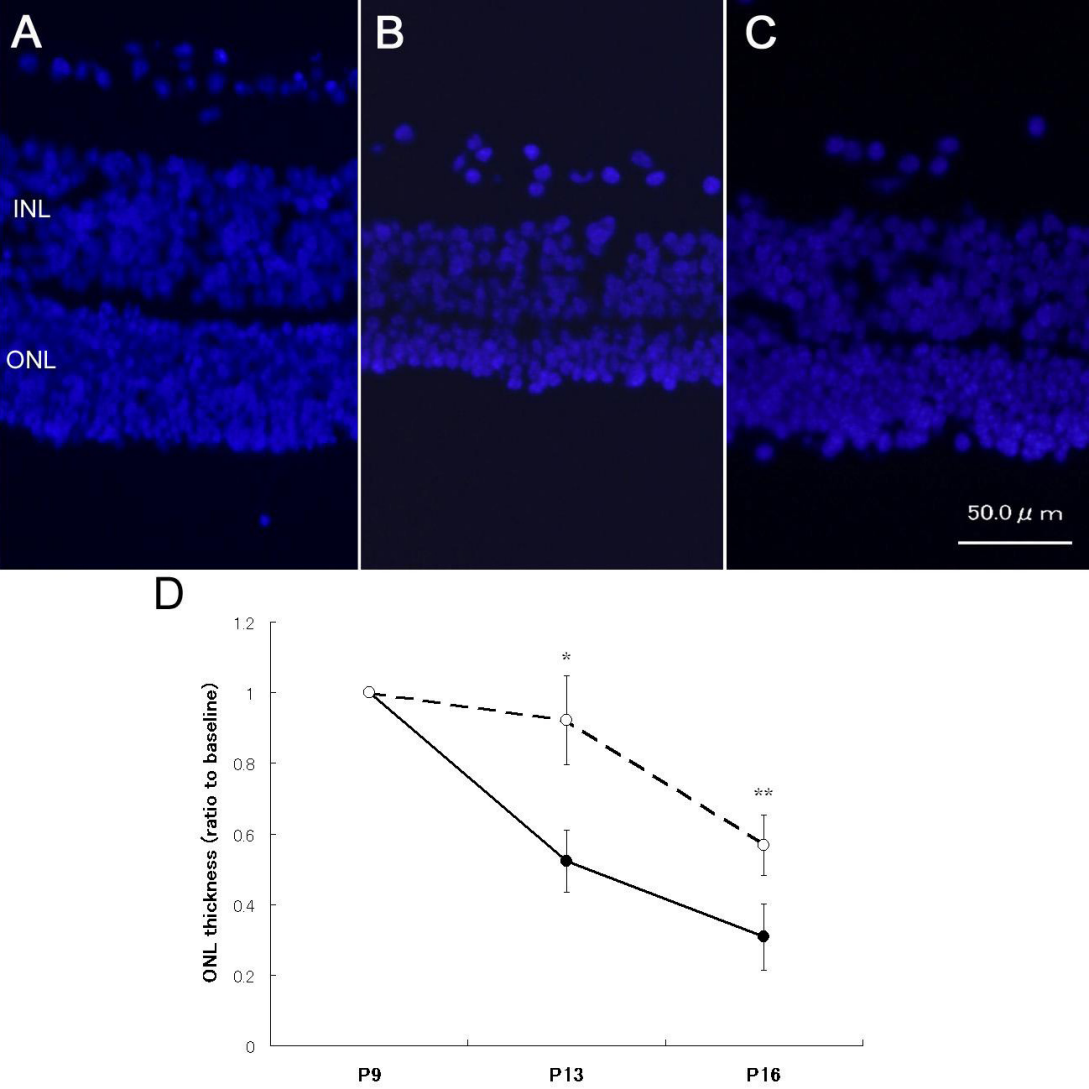
Histological changes in retinal explants. **A**: The morphology of retinal explant at baseline (p9) is shown. **B**: In untreated retinas from rd1 mice (P16), ONL thickness was remarkably decreased. **C**: ONLs were preserved in retinas (P16) transfected for *ASK-1* siRNA-2. **D**: The thickness of ONLs in untreated retinas of rd1 mice decreased with time while the ONLs from retinas transfected for siRNA-2 were significantly preserved. Filled circles indicate control rd1 mouse retina, and open circles represent rd1 mouse retina transfected for siRNA-2. Data are presented as the average±SD of three independent experiments. Asterisk (*) indicates p<0.05, and double asterisk (**) represents p<0.005 compared with control.

ONL thickness decreased markedly during P9 to P16 in retinal explants from rd1 mice (Figure 5). The transfection of siRNA-2 markedly preserved the ONL thickness in rd1 mice. In semiquantitative analyses, ONL thickness in retinas transfected for siRNA-2 was preserved significantly compared with untreated control retinas from rd1 mice (p=0.012 at P13, and p=0.0021 at P16).

### Apoptotic cells in retinas

**Figure 6:**
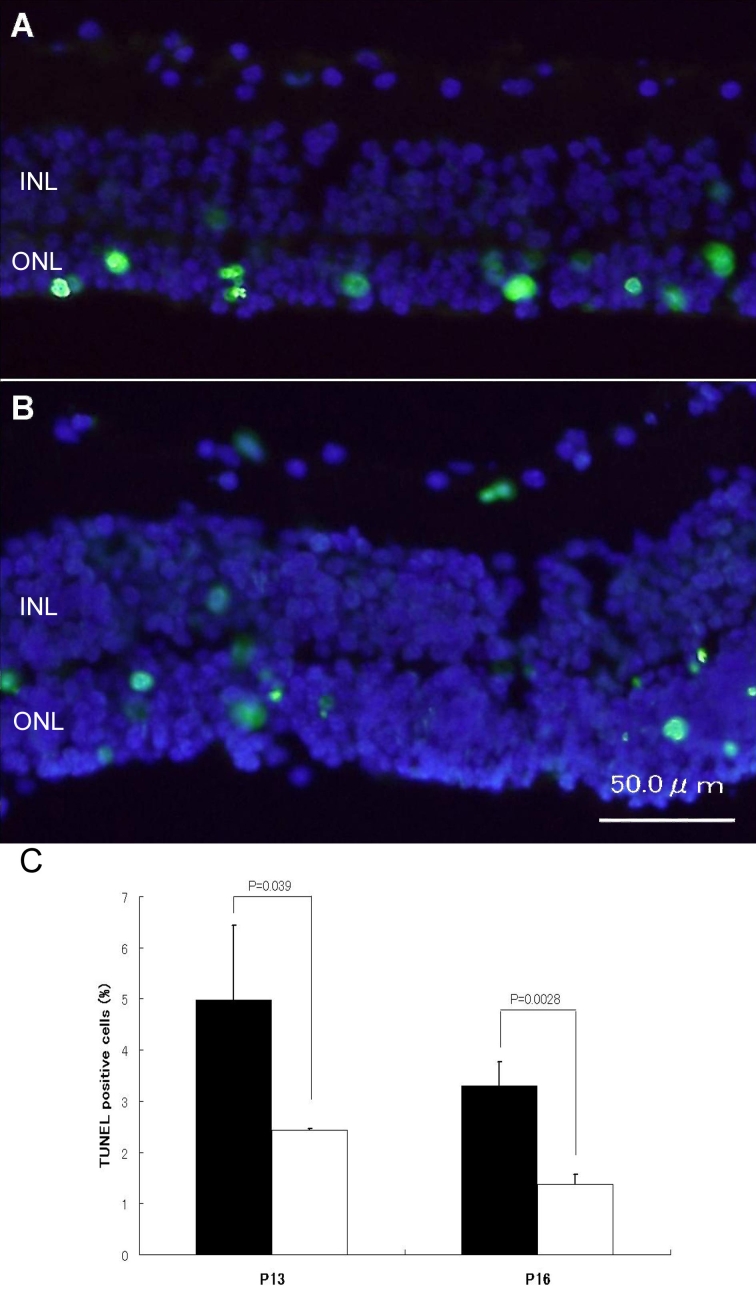
TUNEL-positive cells. **A**: In untreated retinas from rd1 mice, almost all TUNEL-positive cells were found in ONLs while few were found in INLs. **B**: There were few TUNEL-positive cells in retinas transfected for *ASK-1* siRNA-2. **C**: The transfection of *ASK-1* siRNA-2 significantly decreased TUNEL-positive cells compared with untransfected retinas from rd1 mice on day 4 (P13) and day 7 (P16). Filled column denotes control rd1 mouse retina, and open column indicates rd1 mouse retina transfected for siRNA-2. Data are presented as the average±SD of three independent experiments.

Most TUNEL-positive cells were found in the ONL, while few were in the INL in untreated controls and siRNA-2 transfected retinas from rd1 mice. There were fewer TUNEL-positive cells in siRNA-2 transfected retinas than in untreated controls (Figure 6). The mean ratio of TUNEL-positive cells to total ONL cell counts in untreated control retinas of rd1 mice was 5.0% at P13 and 3.3% at P16. Ratios in retinas transfected with siRNA-2 were 2.4% at P13 and 1.4% at P16. The ratio of TUNEL positive cells to total ONL counts in siRNA-2-transfected retinas was about 48% of that seen in untreated retinas at P13, decreasing further to 42% at P16, which was statistically significant (p=0.039 at P13 and p=0.0028 at P16).

## Discussion

In this study, we confirmed that RNA interference is possible in a retinal explant. Further, we demonstrated that ASK-1 is mainly expressed in ONL cells in rd1 mice and plays an important role in inducing the apoptosis of photoreceptors.

Photoreceptor degeneration is known to occur due to various changes to molecules in signal transduction cascades, dysfunctions in energy metabolism, oxidative stress in outer retinas, or disturbances of phagocytic processes by RPE [28]. In the rd1 mouse, a mutation in the enzyme cGMP phosphodiesterase causes a remarkable elevation of cytoplasmic Ca^2+^ [6], which activates multiple pathways of apoptosis including the activation of Ca^2+^ dependent proteases, mitochondrial stress accompanied with the generation of ROS, and ER stress [7-11]. These events are followed by the activation of the ASK-1-JNK-c-Jun pathway (Figure 1) [12,16,18,19,29-31]. The continuous elevation of Ca^2+^ levels in retinas from rd1 mice from P9 to P13 was previously reported [10] to correspond with a gradual increase in the expression of *ASK-1* mRNA, as found in the present study. Moreover, the expression of *ASK-1* mRNA was increased in retinal explants of Bl6 mice after P11, three days from the start of organ culture. It is likely that the oxygen tension in normal atmosphere (about 21%) is high enough to cause oxidative stress in cultured retinal explants and induce *ASK-1* [32].

RNA interference techniques are used in vitro to silence specific genes; Palfi et al. [21] applied RNA interference to knock down rds-peripherin in retinal organotypic culture. Zacks et al. [22] demonstrated that RNA interference for Fas can work in retinal organ cultures to rescue photoreceptors from apoptosis. Lingor et al. [23] applied anti-Apaf-1 and anti-c-Jun siRNA into vitreous cavities of rats and successfully rescued retinal ganglion cells from axotomy-induced apoptosis. Since c-Jun is directly downstream of the ASK-1-JNK apoptosis pathway, the aforementioned findings are consistent with our results. In our study, siRNA was successfully incorporated in retinal explants, and siRNA for *ASK-1* silenced *ASK-1* mRNA expression. Considering that previous reports of RNA interference in vivo or ex vivo resulted in about 50%–80% suppression of target genes [30,33-35], it was likely sufficient that we achieved more than 70% suppression of target genes in retinal explants. The immunoreactivity of ASK-1 was predominantly found in several ONL cells that represented nuclei of photoreceptor cells, which suggests several stresses are present in photoreceptors from rd1 mice to activate ASK-1. It is not clear why only the most distal cells in ONL expressed ASK-1, but it is possible that the increased oxygen tension in the ONL accelerates the expression of ASK-1 [36]. In contrast, *ASK-1* mRNA was found predominantly in inner retinal cells in ischemic retinal injury models that cause strong oxidative stress in inner retinas [29]. ASK-1 immunoreactivity was undetectable in retinas treated with siRNA for *ASK-1*, which means that ASK-1 immunoreactivity in cultured retinas from rd1 mice was possibly regulated by RNA interference. Although the ONL thickness in untreated retinas from rd1 mice was markedly decreased at P16 compared with that at P9, the ONL thickness of retinas transfected with siRNA for *ASK-1* was preserved significantly over the same course. However, all retinal explants tended to decrease in thickness including ONLs in the present study, probably due to the influence of organ culture conditions [37]. This finding was supported by TUNEL assays showing that TUNEL-positive cells were significantly fewer in retinas transfected with siRNA for *ASK-1* at four days and seven days of incubation compared with untransfected control retinas. These findings suggest that ASK-1 is an important molecule for the apoptosis of photoreceptors in the rd1 mouse. However, the distribution of TUNEL-positive cells was not matched to that of ASK-1-positive cells, which suggested the existence of other apoptosis pathways independent of ASK-1.

Kim et al. [38] attempted to rescue photoreceptors from rd1 mice by inhibiting calcium ion dependent proteases or oxygen radicals, but this approach failed to halt apoptosis of photoreceptors. Other studies using p53 or p75NTR knockout rd1 mice did not stop photoreceptor apoptosis [39,40]. In contrast, increasing glutathione transferase levels, adding growth factors such as CNTF with BDNF, or inhibiting poly (ADP-ribose) polymerase promotes rescued rd1 photoreceptors [41-43]. These facts indicate that the mechanism of apoptosis in the rd1 mouse is quite complicated and multiple pathways may work simultaneously. Although apoptosis may occur by several signaling cascades, ASK-1 may be a good target, since it is involved in most stress-induced apoptosis pathways [29-31]. However, downstream signals of ASK-1 are unclear in photoreceptor apoptosis in the rd1 mouse. Harada et al. reported that p38 MAP kinase expression was suppressed in the retina of ASK-1 knockout mice compared with control mice after ischemic retinal injury, while JNK expression was unchanged [29,44]. Further investigations will be needed to determine the complete mechanism of photoreceptor apoptosis in the rd1 mouse

Of course, possible side effects from inhibiting ASK-1 should be considered. A previous report demonstrated that retinal structures and some cell death during development were normal in ASK-1 knockout mice [29]. Interestingly, the knockouts were less susceptible to ischemic injury, and the number of surviving retinal neurons was significantly increased compared with that in wild-type mice. Another problem requiring a solution is the discovery of effective in vivo retinal drug delivery systems for siRNA. Since the in vivo duration of a single administration of siRNA is thought to be one to four weeks [45,46], repeated treatment is required to maintain healthy photoreceptors over time. Chemical modification of siRNA may improve the stability and lengthen in vivo effects [46].

In conclusion, RNA interference of *ASK-1* may become a preventative strategy for retinal degeneration associated with mutations in the cGMP phosphodiesterase beta-subunit. However, further investigations are needed to define the complete effects of *ASK-1* inhibition in vivo.
